# Administrator Perspective on Joy in the Workplace and Worker Retention

**DOI:** 10.1093/geroni/igaf122.634

**Published:** 2025-12-31

**Authors:** Sandi Lane, Darren Liu

**Affiliations:** The University of North Carolina at Charlotte, Charlotte, North Carolina, United States; West Virginia University, Morgantown, West Virginia, United States

## Abstract

Long-term care administrators faced unprecedented challenges during the COVID-19 pandemic while striving to provide quality care and maintain staff morale. Our qualitative study examined the experiences of 21 administrators from North Carolina (n = 15) and Pennsylvania (n = 6) through semi-structured interviews. The research revealed that serving as an administrator during the pandemic was emotionally draining, stressful, and challenging. Communication and relationship-building emerged as the most valuable competencies for effective leadership. Administrators who fostered collaboration among staff and residents positively influenced workplace culture and enhanced problem-solving capabilities. The psychological well-being of staff was directly linked to support mechanisms, particularly caring relationships within the facility. Despite the difficulties, 18 of the 21 participants remained in their roles, describing their work as “a calling,” while three administrators left the field. Innovative communication strategies helped combat isolation, including virtual visits with colleagues, daily voicemail updates, and social media use to reassure families. For administrators, feeling valued and part of a team proved crucial in managing the challenges of caring for residents and staff during this crisis. The study also highlighted the importance of enhancing the public image of the long-term care industry to drive meaningful change and improve experiences for both staff and administrators.

